# Microbes and Antibiotics: An annotated selection of World Wide Web sites relevant to the topics in *Microbial Biotechnology*

**DOI:** 10.1111/1751-7915.12088

**Published:** 2013-10-11

**Authors:** Lawrence P Wackett

**Affiliations:** Department of Biochemistry, Molecular Biology & Biophysics, BioTechnology Institute, University of MinnesotaSt. Paul, MN, 55108, USA

## List of antibiotics: Wikipedia

### The http://en.wikipedia.org/wiki/List_of_antibiotics

This is an excellent starting point for research on antibiotics. The site contains a large list of antibiotics that is arranged into different structural classes.

## Antibiotics: actions, origins and resistance

### http://forms.asm.org/microbe/index.asp?bid=28714

This review describes a book that provides an excellent background on the types of antibiotics, their synthesis and their mechanisms of action.

## Timeline of antibiotics: Wikipedia

### http://en.wikipedia.org/wiki/Timeline_of_antibiotics

This timeline shows the dates of introduction of new antibiotics. It should be noted that the major classes of antibiotics were largely discovered more than 40 years ago and that many recent antibiotics are elaborations on existing antibiotic structures.

## Are we running out of antibiotics?

### http://www.thedailybeast.com/newsweek/2010/12/07/are-we-running-out-of-antibiotics.html

This article for a general audience lays out the idea that the discovery of new types of antibiotics has fallen off in recent years and that antibiotic resistance is increasingly a problem in the practice of medicine.

## Chronic, low antibiotic dosage and obesity

### http://www.nature.com/nature/journal/v488/n7413/full/nature11400.html

Long-term, sub-therapeutic doses of antibiotics can increase weight gain for a given dietary intake. This has positive implications for purposeful increase in the weight of farm animals but negative consequences for humans where obesity is a major current health concern.

## Antibiotics: open access journal

### http://www.mdpi.com/journal/antibiotics

This link is for the open access journal *Antibiotics*.

## Silver makes antibiotics more effective

### http://www.nature.com/news/silver-makes-antibiotics-thousands-of-times-more-effective-1.13232

Silver compounds have long been known to have antimicrobial activity, but new research suggests that silver acts synergistically with many common organic antibiotics and thus could help against antibiotic resistance issues.

## Antibiotics and innovation: Pew Charitable Trusts

### http://www.pewhealth.org/projects/antibiotics-and-innovation-project-85899367216

The Pew Charitable Trusts has invested in research and societal issues related to antibiotic resistance and new antibiotic development.

## Antibiotics may harm mitochondria

### http://www.the-scientist.com/?articles.view/articleNo/36329/title/The-Downside-of-Antibiotics-/

A recent report suggests that antibiotics may damage the mitochondria in animal cells. This is interesting in the context of human health and evolution because mitochondria are thought to derive from prokaryotic cells.

## Bacterial resistance to antibiotics

### http://textbookofbacteriology.net/resantimicrobial.html

These pages from Ken Todar's online microbiology textbook give an excellent overview of antibiotics.

## Antibiotics: Sigma-Aldrich

### http://www.sigmaaldrich.com/chemistry/chemistry-products.html?TablePage=14572853

This catalogue page provides links to lists of antibiotics, information on chemical classes and the mechanism of action of antibiotics.

## How antibiotics work still controversial

### http://www.scientificamerican.com/article.cfm?id=antibiotics-more-mysterious-than-appear

This popular science article discusses some of the controversies concerning the mechanisms of action of antibiotics.

## Questioning multiple-antibiotic strategies

### http://www.plosbiology.org/article/info%3Adoi%2F10.1371%2Fjournal.pbio.1001540

There is a common perception that combining antibiotics for medical treatment is the best way to protect health and prevent antibiotic resistance. The present paper uses computer simulations and experiments to suggest otherwise.

## Streptomyces: Wikipedia

### http://en.wikipedia.org/wiki/Streptomyces

The major antibiotic-producing genus is the bacterial genus *Streptomyces*.

## Streptomyces: Microbe Wiki

### http://microbewiki.kenyon.edu/index.php/Streptomyces

This Microbe Wiki site discusses *Streptomyces* and the antibiotics they produce.

## New way to improve antibiotic production

### http://www.bbsrc.ac.uk/news/industrial-biotechnology/2013/130618-pr-improve-antibiotic-production.aspx

This report describes a discovery in which an antibiotic provides a regulatory signal for a bacterium to produce more antibiotics. This could be used as a strategy to increase the industrial production of antibiotics.

## Soil bacteria eat antibiotics

### http://www.the-scientist.com/?articles.view/articleNo/33609/title/Soil-Bacteria-May–Eat–Antibiotics/

This popular news article describes research in which bacteria can use antibiotics as a carbon and energy source. The danger is that high-level antibiotic degradation activity might spread among microbial populations.

## The business of antibiotics: Forbes

### http://www.forbes.com/sites/stephenbrozak/2013/05/07/the-antibiotic-bubble-a-quest-for-continued-antibiotic-effectiveness/

This article deals with the business aspects of antibiotics and antibiotic resistance.

## Speeding up the introduction of new antibiotics

### http://www.nytimes.com/2013/06/03/health/experts-debate-plan-to-speed-antibiotic-development.html?pagewanted=all&_r=0

There are rising concerns that businesses do not have suitable incentives to develop new antibiotics and programmes are emerging to stimulate new research on antibiotics.

